# IoT Platforms and Security: An Analysis of the Leading Industrial/Commercial Solutions

**DOI:** 10.3390/s22062196

**Published:** 2022-03-11

**Authors:** Giancarlo Fortino, Antonio Guerrieri, Pasquale Pace, Claudio Savaglio, Giandomenico Spezzano

**Affiliations:** 1 DIMES, University of Calabria, Via P. Bucci, 41C, 87036 Rende, Italy; giancarlo.fortino@unical.it (G.F.); p.pace@dimes.unical.it (P.P.); 2 Institute for High Performance Computing and Networking-National Research Council of Italy (ICAR-CNR), Via P. Bucci, 8/9 C, 87036 Rende, Italy; antonio.guerrieri@icar.cnr.it (A.G.); giandomenico.spezzano@icar.cnr.it (G.S.)

**Keywords:** Internet of Things, IoT platform, reference architecture, IoT security

## Abstract

For simplifying and speeding up the development of the Internet of Things (IoT) ecosystem, there has been a proliferation of IoT platforms, built up according to different design principles, computing paradigms, technologies, and targets. This paper proposes a review of main examples populating the wide landscape of IoT platforms and their comparison based on the IoT-A reference architecture. In such a way, heterogeneous IoT platforms (both current and future) can be analyzed regardless of their low-level specifications but exclusively through the lens of those key functionalities and architectural building blocks that enable the interplay among devices, data flow, software, and stakeholders within the IoT ecosystem. Among these, *security by design* (i.e., the inclusion of security design principles, technology, and governance at every level) must be integrated into every tier, component, and application to minimize the risk of cyber threats and preserve the integrity of the IoT platforms, not only within individual components but also for all the components working together as a whole.

## 1. Introduction

The Internet of Things (IoT) [1,2], at its highest realization, is an ecosystem of heterogeneous devices (sensors, actuators, gateways, smart gadgets, RFID/NFC tags, edge nodes, cloud servers, etc.) and software components (API, middlewares, ontologies, digital libraries, digital twins, etc.) that seamlessly cooperate and interact with human users to provide advanced and contextualized cyber-physical services [3]. Indeed, the IoT is widely considered a technological cornerstone for a plethora of emerging smart applications (smart factory, smart city, smart vehicles, smart buildings), as well as a key enabler for well-established domains like ambient, assisted living, domotics, industrial automation, etc.

Due to the IoT’s complexity, many frameworks and platforms have been recently proposed for supporting developers in architecting, programming, and exploiting such articulated bundles of hardware, data, and applications [4,5,6]. There has been a proliferation of IoT platforms because these are featured by a shorter distance to the implementation phase, good flexibility, and higher usability with respect to IoT frameworks [1]. In more detail, IoT platforms allow achieving more specific operational outcomes with reduced time-to-market, risks, and development costs, by delivering (in a secure way) key functionalities like applications enablement, data flow design, devices, and connectivity management. As result, instead of focusing on the lower levels of the technology stack, IoT platforms allow for a greater concentration of resources in value-add applications, thus being extremely attractive both for individual developers and small/medium/large enterprises. As a matter of fact, an increasing number of IoT platforms exist at the state-of-the-art, both with general- and domain-specific purposes (e.g., IoT platform for COVID-19 prevention and control [7], IoT energy platforms [8], IoT platforms for smart city [9]), and they are constantly updated to support the latest technology solutions (edge computing, blockchain, digital twins, just to name a few) [10,11]. Definitely, IoT platforms will enable an enhancement in the way we work and live as long as they ensure the integrity and confidentiality of IoT solutions and data while mitigating cybersecurity risks [12].

The landscape of IoT platforms results increasingly wide and heterogeneous: today, this market comprises more than 600 vendors (however, a small percentage of them, around 10%, have billion-dollar revenue streams and can be considered more than a start-up) predominantly targeting business/enterprise (especially manufacturing and industrial IoT solutions) and, to a lesser extent, consumer segment (domotics) [13]. Likewise, there exists a good number of survey works that analyze the scenario of IoT platforms with different approaches and goals. A shortlist of key functionalities (device management, connectivity, data analytics, simulation, and visualizations tools) is used to compare IoT platforms able to support large-scale deployment [14,15], cloud-based systems [16], do it yourself (DIY) projects [17] or industrial IoT scenarios [18]. High-level features like scalability, stability, interoperability, pricing models, etc., instead, have been recognized as discriminant factors in [19,20,21] to drive the adoption of an IoT platform from an enterprise perspective. In [22], instead, a systematic and more comprehensive review of IoT platforms based on their supported development phases (modeling, analysis, design, implementation, testing) is provided, in order to explore the engineering lifecycle processes. Only two surveys, namely [23,24], provide a reference architecture (RA) as a comparison framework for systematically analyzing so much different IoT platforms with a suitable level of detail. This is a wise and effective choice: indeed, a RA allows to simultaneously consider, regardless of low-level specifications, the aforementioned high-level functionalities of IoT platforms, their main building blocks, and their key design choices.

Along such lines, in this paper, we provide a review of the main IoT platforms, and we compare them through the IoT-A RA [25]. Indeed, differently from [23,24], we do not introduce the umpteenth RA (see [26] for a detailed list) but we consider the most acknowledged one, widely referred and globally considered the baseline for the other successful RAs presented over the years, like AIOTI, IEEE P2413, WSO2, etc. The exploitation of the IoT-RA provides generality to the provided contribution and soundness to the performed analysis, thus eventually steering the reader in the wide landscape of IoT platforms. In particular, in contrast with other prior surveys and to extend our previous work [27], in this paper, we shed light also on novel paradigms (e.g., edge computing, digital twin) and approaches (e.g., trustworthiness, edge intelligence), as well as on the security topic within the IoT platforms. Security, considered in past years as an afterthought in IoT projects (security features are commonly cut from initial designs to accommodate additional device functionality) and as an option for end-users (poor awareness of cost and implications of IoT security breaches), has recently elevated its importance within IoT platforms [28]. As a matter of fact, in 2020, companies increased by 40.3% the spending on IoT security over data and devices [29], motivated by the visibility provided by the media to IoT cybersecurity incidents. Hence, security best-practices such as employing a secure boot process or using unique identity keys and mapping the attack surface are now well-established in the IoT platform landscape, along with other mechanisms surveyed in the following. These cover multiple levels and fuse together important security features across the whole IoT platform stack since the development of secure end-to-end IoT solutions requires a holistic approach.

The rest of the paper is organized as follows. In Section 2 we report the IoT-A RA that considers the main functionalities and building blocks of an IoT platform. According to such RA, we review the main IoT platforms in Section 3 by providing first an introduction and then zooming in on their main security aspects. A summary and a discussion about the provided analysis are drawn in Section 4, while final remarks conclude the paper.

## 2. IoT-A Reference Architecture

The IoT-A project [25], funded by European FP7-ICT, was aimed at creating an architectural reference model for interoperable and scalable IoT ecosystems as well as at outlining key building blocks, development principles, and guidelines for the technical design of their protocols, interfaces, and algorithms. One of the main advantages of the IoT-A architectural model is the creation of a single global ecosystem of things that communicate seamlessly with each other to support the advanced digital services necessary for the digital transformation of industrial and business processes.

**Figure 1:**
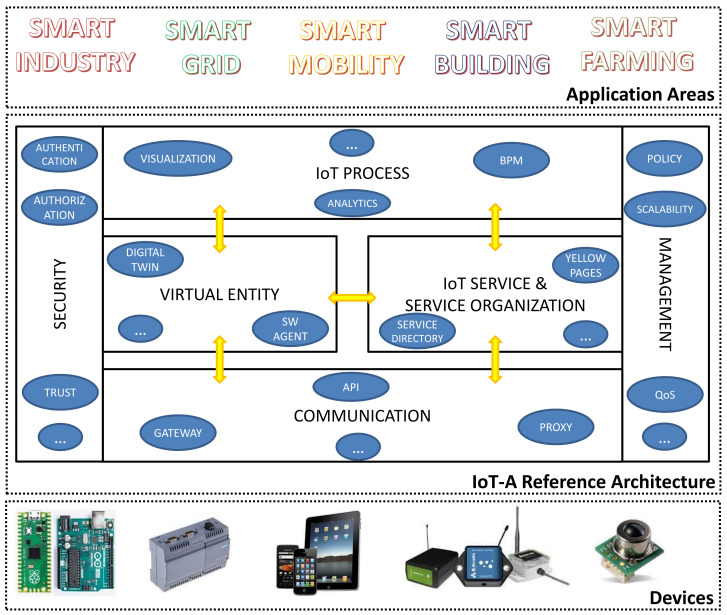
IoT-A reference architecture: architectural layers and main components/functionalities.

The resulting IoT-A RA mainly consists of six layers (also depicted in Figure 1):

The communication layer considers the variety of communication schemes (both local and remote) derived from the many technologies belonging to IoT systems and it provides a common interface to the IoT Service layer. Here, a set of protocols as well as hardware and software solutions (gateway, proxies, APIs, etc.) for enabling the communication within and between IoT systems find the place, making this layer fundamental for providing the basic interconnectivity.

The *virtual entity layer* provides the solutions for interacting (e.g., discovering, managing, shadowing, looking up services) with the physical IoT systems’ components through cyber counterparts such as digital twins, virtual entities, software agents, etc. All these virtual aliases allow storing the history of their related asset, retrieving and updating information, monitoring the status and properties, by means of remote high-level interfaces.

The *IoT service and service organization layer* deals with IoT Service, a primary abstraction representing the entry point for the functionalities provided by the IoT systems and their components. Therefore, being linked to all the other layers, it is central within the RA, also providing utilities related to services discovery, look-up, name resolution, composition, and orchestration.

The *IoT Process Layer* is in charge of integrating conventional process management systems with the IoT system, by providing the functional concepts, interfaces, and tools necessary to augment traditional (business) processes. Therefore, this layer provides an environment for the modeling of the overall IoT system as well as for analytics and visualization tools for processing and displaying the data. Other important functionalities like simulation might be placed at this layer to provide full support to the IoT process execution.

The *security layer* ensures the security and the privacy of an IoT system by means of well-established mechanisms of authorization, authentication, key exchange and management, trust and reputation, etc. Since security and privacy are concerns of paramount importance for all the components of IoT systems and by-design solutions are typically necessary to achieve a satisfactory degree of protection, this layer is transversal to all the previous ones.

The *management layer* combines all functionalities that are needed to govern an IoT system like fault handling, configuration, accounting, and performance. Solutions aimed at automatic load balancing, horizontal and vertical scalability, data backup, usage policy, etc. are addressed here and are designed for being as automated as possible, thus minimizing the need for human intervention. As for the security layer, this is also a cross-layer.

Given this brief description of IoT-RA and by extracting its essentials, concrete yet heterogeneous IoT platforms can be effectively compared regardless of their low-level technical specifications or specific targets, as done in the following.

## 3. State-of-the-Art Analysis

This section will overview those IoT platforms most recurring in both commercial [13] and academic [4,5,6] studies, most widely adopted and hardware-agnostic (to this end, we have not considered, for example, Cisco IoT which is purposely designed to work almost exclusively with Cisco IoT devices), and which we consider particularly interesting based on our experience in the field [1,12]. Obviously, given the dynamicity and wideness of the IoT landscape (where more than 600 platforms currently exist) [30]), the provided overview does not want to be exhaustive; conversely, this work aims to compare main IoT platforms to date available, regardless of their heterogeneity and specificity, but in the light of their basic, advanced or peculiar contributions to the key functionalities expected at the different IoT-RA layers. The main highlights of this comparison are reported in Table 1 and they will be further commented in Section 4. A separate analysis will be provided, for each platform, regarding the security aspects and the highlights will be reported in Table 2. Please note that some cells are blank since every IoT platform provides information with a different degree of detail or sometimes this is not available at all.

### 3.1. Siemens MindSphere

*MindSphere* [31] is the Siemens IoT platform declared as the leading “industrial IoT as a service” solution. It has been used, for example, for proactive maintenance and anomaly detection in the field of smart manufacturing. Moreover, it has been successfully applied in several scenarios, both industrial and civil, to reduce energy and water wastage.

MindSphere uses advanced analytics and AI to make running IoT solutions from the edge to the cloud with data from connected products, plants, and systems to optimize operations, create better quality products, and deploy new business models. It allows to quickly collect, monitor, and analyze data in real-time and offers insights that improve efficiency and profitability. MindSphere can run on different cloud infrastructures such as AWS, Azure, and Alibaba. In particular, the MindSphere Gateway and the MindConnect Integration APIs provide great support for the communication layer as well as the Siemens Digital Twins and the MindSphere Predictive Learning Analytics for the IoT Service and Process Layers.

#### Security in MindSphere

The platform allows customers to confidently operate in a secure cloud environment. Data protection throughout the lifecycle, from connecting devices and data retention to decommissioning is the main mission achieved through the implementation of security-by-design concept. MindSphere follows the International Organization for Standardization (ISO) 27001 Information Security Management System Framework and it is certified for International Electrotechnical Commission (IEC), secure development lifecycle (SDL). This standard defines requirements related to cybersecurity for products intended for use in the industrial automation and control systems environment as well as best practices for information security management processes. Identity management and access control are always implemented through JSON Web Token (JWT) checks. Authentication is implemented through multifactor authentication (MFA) to confirm the identities of users trying to access the system by following international industry standards to choose the right password according to the required strength. The fine-grained authorization process comes from MindSphere APIs to validate authorized calls depending on the context of the application and the user permissions. All communication from the client to MindSphere through public endpoints is secured through Transport Layer Security (TLS) v. 1.2, and Reliable x509 certificates are used from the Siemens Trust Center, which is trusted by the European Telecommunications Standards Institute (ETSI) and the Certification Authority Browser Forum. To guarantee a secure, cost-efficient, and easy device connectivity, the MindSphere platform implements the so-called MindConnect devices embedding a unique identification number, thus, only valid MindConnect devices from Siemens are onboarded to MindSphere.

### 3.2. IBM Watson

*IBM Watson* [32] was initially conceived as a question answering computing system developed to apply, to such a field, several advanced technologies like information retrieval, knowledge representation, advanced reasoning, and machine learning. Now, Watson includes a set of business-ready tools, applications, and solutions, designed to reduce both the costs and the hurdles of AI adoption while optimizing outcomes and responsible use of AI.

Among the offered tools, the *Watson IoT Platform* [33] allows the use of fully managed, cloud-hosted services with capabilities for device registration, connectivity, control, rapid visualization, data storage, and asset monitoring, especially through the IBM Cloud BLUEMIX, based on Cloud Foundry and IBM Maximo. It also provides auto-load balancing mechanisms and automatically scaling granularity with Iaas, Paas, and SaaS models. All of this allows the platform to provide great support to both IoT service and IoT process layers. The built-in support for simulation tools is a distinctive feature of the Watson IoT Platform belonging to the virtual entity layer. Moreover, it fully supports the blockchain for data exchanging and allows users to take full advantage of the Watson cognitive APIs applied to the IoT world.

The use of the Watson IoT Platform includes a wide range of application areas that span from healthcare to business automation and from advertising to risk assessment. Moreover, it is used with great success in the fields of smart manufacturing, smart agriculture, and smart buildings.

#### Security in Watson

The platform is certified under the aforementioned ISO 27001 standard [34] document. IoT information is secured through the use of HTTPS communication protocol for both browser-based GUI and REST APIs with a certificate that is signed by DigiCert in order to set a trusted connection to the genuine platform service. Moreover, the access to the web-based GUI is authenticated by a unique IBM identification code. Device and application credentials are secured through an authentication token that is salted and hashed in order to guarantee a strong authentication level. Each device is connected to the platform by using TLS v.1.2 security by default, which ensures that devices can connect only by using a secure, encrypted channel. To prevent devices from being able to impersonate another device, once authenticated, devices are only authorized to publish and subscribe to a restricted topic space and the authentication credentials that are provided by the client dictate to which device the topic space is scoped by the platform service.

### 3.3. Amazon Web Services

*Amazon Web Services* (AWS) [35,36] is the biggest player in the cloud market [37]. It is a platform of web services offering different instruments for computing, storing, and networking. All of this is provided at different levels of abstraction. All the services are available through common Internet protocols although both APIs and gateway can be customized. The most used services comprehend *EC2*, which has been developed to provide virtual machines, and *S3*, which provides storage. A very useful service offered by AWS is *AWS IoT* [38] which allows to connect IoT devices and collect, store, and analyze their data. AWS clients can choose to use one of many data centers scattered around the world or, through AWS IoT Greengrass, to move data processing and analysis as close to the end-point as necessary. AWS IoT relies on several components useful to comply with specific functions, for example, modules for data handling and analytics (e.g., Amazon S3, Kinesis, Lambda) need to be separate. In particular, among these, the *AWS IoT Device Management* gives support for both the IoT Service and the management layers thus enabling the organization/monitoring IoT devices and implementing priority-based SLA, and the *AWS IoT Core*, which allows users to easily connect devices and characterizes the AWS IoT’s communication layer.

AWS IoT is widely used by important industrial players for the realization of industrial applications. Moreover, it is adopted for the realization of several smart agriculture and smart city scenarios, with a particular emphasis on smart home due to the many home assistant and multimedia devices from the same brand.

#### Security in AWS IoT

In AWS IoT, each connected device or client must have a specific access credential, and all traffic to and from AWS IoT is sent securely over TLS v. 1.2 [39]. Furthermore, AWS cloud security mechanisms protect data as it moves between AWS IoT and other AWS services. Authentication takes place at the TLS layer through validation of the X.509 certificate chain; the same method is used by the browser when visiting an HTTPS URL. AWS IoT supports three types of identity principles for device or client authentication, namely X.509 client certificates, IAM (Identity and Access Management) for users/groups/roles, and Amazon Cognito identities. All data sent to AWS IoT is sent over a TLS connection using MQTT, HTTPS, and WebSocket protocols, making it secure by default while in transit. Data sent to and from device advisor are encrypted in transit.

### 3.4. SAP IoT

*SAP* [40] is one of the most important producers of software for the management of business processes. It aims to develop solutions enabling effective data processing and information flow across organizations. SAP software provides many business functions with a whole single view. This allows companies to manage complex business processes with only one instrument.

The *SAP Internet of Things (SAP IoT)* [41] solution uses SAP to tackle the IoT world so offering capabilities to address Industrial IoT (IIoT) use cases. Such matching allowed SAP to be named as Leader for Worldwide Industrial IoT Platforms and Applications in Manufacturing by IDC MarketScape. Besides the IIoT, SAP IoT is successfully used in the agri-food sector, to rationalize and manage retail companies, and to realize predictive maintenance applications.

SAP IoT enables users to re-design business processes with embedded IoT services and data, giving high support to the IoT Process Layer. SAP IoT exploits cloud services for building IoT applications, enriches IoT data with business context, and provides analytical services with live integration to *SAP Analytics Cloud*. Moreover, it relies on scalable device management services to fully support the management layer. The SAP IoT simulator uses sensory and machine data that is collected and analyzed on the *SAP HANA cloud platform*. A certified catalog of third-party connectors and APIs make SAP IoT greatly interoperable with the other main IoT platforms, both commercial and industrial.

#### Security in SAP IoT

All device connectivity APIs use the TLS protocol to secure the communication between the client and the server [42]. X.509 certificates are used in many internet protocols, including TLS/SSL, which is the basis for HTTPS, the secure protocol for browsing the Web. All server certificates are issued by well-known and generally trusted certificate authorities, for example DigiCert. From the network communication point of view, the platform uses standard mechanisms to establish secure links among its components; in particular: (i) devices can connect to the IoT gateway cloud (MQTT or REST) through a secure TLS version 1.2, where client certificate authentication is in place; (ii) security between devices and the IoT edge platform depends on the protocol implemented by the devices. The specific IoT edge platform implementation is in charge of leveraging the protocol security mechanism to guarantee end-to-end security from devices up to applications. Since the purpose of SAP IoT is to enable customers to build applications, SAP partners, as well as SAP customers who build applications based on SAP IoT, and who consume SAP IoT services, are in charge of ensuring the overall data protection and privacy compliance.

### 3.5. PTC Thingworx

*ThingWorx* [43] from *PTC* [44] is one of the first IoT platforms appositely conceived to address the industrial IoT world. It is widely used in the field of manufacturing for the real-time control of productivity and for the integration of data from several production sites. Moreover, it is successfully used in several industries for predictive maintenance.

The architecture proposed in ThingWorx incorporates modular functionalities that simplify the development of applications that can be quickly and easily implemented by merging pre-built modules (e.g., connectors for exchanging data from heterogeneous devices and systems or UI elements to build the desired GUI) or third-party services for resource management (e.g., load balancing, automatic scaling) from Amazon AWS, Microsoft Azure, and other public device clouds, as part of ThingWorx’s Open Platform Strategy. Such capability allows to successfully provide support to the management layer. Its strongest point, however, is the *Thingworx Analytics* module, purposely designed for predictive maintenance and machine learning tasks as well as integrated with the ANSYS simulator, aiming to turn data into actionable intelligence for smarter product design.

#### Security in ThingWorx

ThingWorx is secure by design and offers multiple authentication options to increase the security of IoT applications. The ThingWorx platform includes specific functionalities designed for industrial IoT, taking into account the connectivity, scalability, and security to grow with the business. In particular, any communication is device-initiated, TLS-encrypted, and directed to only one server. Security patches are distributed through our software content management tool, while role-based access controls allow for granular control of things, their data, and the actions available in any specific application. Finally, an active directory provides a single place to manage user and user groups and simplifies access management for enterprise IT administrators. The end-to-end security model (built-in security) simplifies the development and operation of secure IoT solutions. The integrated user administration with role-based access permissions can provide additional security for data, users, administrators, and developers.

### 3.6. Microsoft Azure

Microsoft Azure [45,46] is the general brand name for Microsoft’s cloud-computing created for building, testing, deploying, and controlling applications and services through Microsoft-managed data centers. Azures provides, similarly to IBM Watson and Amazon AWS, mechanisms for auto-load balancing and automatically scaling granularity, beside a plethora of services ranging from software as a service (SaaS), to platform as a service (PaaS), and infrastructure as a service (IaaS). Moreover, it has been built to manage different programming languages, tools, and frameworks, also from different vendors. These features, along with the availability of Windows IoT (formerly Windows Embedded), makes Azure an appealing platform for a wide range of domains (healthcare, retail, manufacturing, energy, logistics, and transportation) and IoT devices (small devices and wearables, kiosks, ATMs, etc.).

*Azure IoT* [47,48] is a set of Azure cloud services that connect, monitor, and control IoT devices. In Azure IoT, an IoT solution is composed of one or more IoT devices and one or more back-end services running in the cloud that communicate with each other. The back-end services gather sensor data and determine how to process and store that data. Such functions are provided, among the others, by the *Azure IoT Central* component, which accelerates the creation of IoT solutions, and reduces the burden and cost of IoT management, operations, and development so giving full support to the IoT process layer. Moreover, above all, Azure IoT allows one to (i) deploy SaaS solutions for IoT with minimal cloud expertise; (ii) customize industry-specific IoT solutions for common IoT scenarios; (iii) dislocate intelligence from the cloud to edge devices (Azure IoT Edge); (iv) use digital models of physical things (*Microsoft Azure Digital Twins*). This last one is a very important and well-implemented characteristic of Azure IoT that allows giving full support to the virtual entity layer.

#### Security in Azure IoT

The “Azure IoT Hub” within the IoT solution accelerators offers a fully-managed service that enables reliable and secure bi-directional communication between IoT devices and Azure services by using per-device security credentials and access control. In particular, the platform supports the following three features: (i) *secure device provisioning and authentication*, with a unique identity key for each device, which can be used by the IoT infrastructure to communicate with the device while it is in operation. The generated key with a user-selected device ID forms the basis of a token used in all communication between the device and the Azure IoT Hub; (ii) *secure connectivity*, with the Azure IoT Hub supporting HTTPS along with AMQP and MQTT, not only for efficiency in terms of resource use but also reliable message delivery. Azure IoT Hub enables secure connection to both IP-enabled and non-IP-enabled devices. IP-enabled devices are able to directly connect and communicate with the IoT Hub over a secure connection using TLS and X.509 protocol. Non-IP-enabled devices are resource-constrained and connect only over short-distance communication protocols, such as Zwave, ZigBee, and Bluetooth. A field gateway is used to aggregate these devices and perform protocol translation to enable secure bi-directional communication with the cloud; (iii) *Secure processing and storage in the cloud*, with the Azure Active Directory (AAD) for user authentication and authorization, so to provide a policy-based authorization model for data in the cloud and enable the easy access management that can be audited and reviewed. Moreover, *Azure Sphere* security service offers an integrated solution to protect IoT devices, operating systems, and cloud services. It adds multiple layers of defense, provides continuous device monitoring, and enables returning compromised hardware components to their safe states.

### 3.7. BOSCH IoT Suite

The *Bosch IoT Suite* [49] is an open-source-based IoT platform specialized in developing, testing, and running scalable IoT services and applications. Such services and applications are actually used to improve productivity across all industries. In particular, Bosch IoT Suite is successfully used for smart agriculture, energy management, logistics, industrial IoT, and smart home.

Great support to the communication layer is provided through the Bosch IoT Gateway and a rich set of local and remote APIs that allow securely connecting several kinds of heterogeneous devices, sensors, microcontrollers, and manage IoT data both at the edge and at the cloud. The Bosch IoT Hub, instead, provides a managed inventory of digital twins for IoT device assets, whose data are normalized, stored, and enriched by the Bosch IoT Insights at the IoT service layer.

#### Security in BOSCH IoT Suite

Bosch IoT Suite offers distinctive components in the security context such as

(i) central management of asset data and secure messaging; (ii) user management, role-based access control and multi-tenancy for IoT applications; (iii) management of large-scale rollouts of device software/firmware updates (wired or over-the-air). Additionally, two important security features pertain the device authentication and transport layer encryption. With respect to the former, Bosch IoT Hub relies on protocol adapters to establish a device’s identity before it is allowed to publish telemetry data or send events; thus, a device presents an *auth-id* as part of its credentials during the authentication process which is then resolved to a device identified by the protocol adapter on successful verification of the credentials. Device authentication can support the basic username and password, the advanced X.509 certificate, or a PSK (pre-shared-key) through a CoAP protocol adapter. With respect to the transport layer encryption, Bosch IoT Hub uses encryption for all connectivity through the TLS protocol for all TCP-based endpoints and DTLS protocol for UDP-based endpoints. Specific protocol adapters have been developed for both TCP-based (AMQP, HTTP, LoRa, and MQTT) and UDP-based (CoAP) connections. Due to security best practices, IoT Hub does not allow potentially insecure protocols like SSL or TLS lower than version 1.2.

### 3.8. GE PREDIX

*GE Predix* [50,51] is an IIoT software platform created with the aim of helping users to develop and deploy intelligent systems for the monitoring and the control of physical devices or whole systems through the Internet. In this direction, it supports heterogeneous data types like asset data (time-series, HMI/SCADA, sensory data, etc.), plant data (e.g., operations, alerts, and key performance indicators from execution systems), and enterprise data (for example, computerized maintenance data from the management system). Predix, which can be deployed at the edge (*Predix Edge*, for local data processing, filtering, container-based applications) or in the cloud (*Predix Cloud*, for large-scale data ingestion, analytics processing, storage), combines a set of different industrial technologies for distributed computing and big data analytics, machine-to-machine communication, and mobility. The *Predix Connectivity* component of the platform offers secure and reliable communication between the devices involved in any application so giving good support to the communication layer. The IoT process layer is implemented, among the others, with a built-in user console, which provides IoT visibility and event management without the need to implement custom applications. Finally, Predix allows users to create digital twins of real elements.

Although Predix can support many kinds of applications for the IIoT, it has been optimized for power generation and the oil, gas, and chemical industries. For these fields, several software and services are available and ready to use.

#### Security in GE PREDIX

The Predix platform is secure by design, providing capabilities such as two-party encryption and supporting end-to-end chain of custody reporting for code and data [52]. Security policies at multiple layers are applied to limit access to GE Digital employees who possess a legitimate business need for such access. Additionally, data are de-identified where needed and transmitted in encrypted form using Transport Layer Security (TLS) and tokenization. Predix is built on a common infrastructure governance model based on ISO 27001/2, NIST 800-53, and FIPS 140-2; in addition, it extends protection to devices beyond the cloud, running mission-critical analytics, the Incident Command System (ICS), manufacturing, and other industrial IIoT and critical infrastructures. Predix supports sophisticated mechanisms to prove identities, create roles across the ecosystem, and effectively authenticate and authorize access while privileged accounts are further contained and managed. Predix supports hardware security modules, key management systems, and public and private key infrastructures for effectively protecting and managing keys. In addition, the Predix platform provides APIs to integrate encryption and data protection with any of the services developed or deployed in the Predix environment.

### 3.9. Hitachi Vantara Lumada

*Lumada* [53,54] from Hitachi Vantara [55] is an IIoT platform that helps customers rapidly create digital solutions. Lumada claims to provide users with, at the virtual entity layer, an advanced support to digital twins and digital twins modeling Furthermore, at IoT process layer, it equips the *Lumada Analytics* for advanced analytics and AI algorithms and the *Hitachi Visualization Suite*, which allows versatile management of an IoT system. Lumada provides secure connectivity with industrial IoT sensors, actuators, and gateways. It is realized in modular components that can be distributed both at the edge and in the cloud so permitting a fully distributed system. Finally, Lumada is open and interoperable in the sense that it can work with any industrial system and any cloud, including with MATLAB and Simulink for modeling and simulating.

Several use cases have been actually built on Lumada whose main fields of application span from smart manufacturing to smart city.

#### Security in Lumada

Very poor information is provided about Lumada security. From developers forums [56] it emerges that Hitachi Vantara Lumada is certified to ISO/IEC 27001 and it uses SSL when communicating with other applications, along with cryptographic keys, X.509 certificate chains, and trusted certificates. However, no further information is available, to the best of our knowledge.

### 3.10. Google Cloud IoT Core

*Google Cloud* [57] is a set of cloud computing services by Google providing users with computing, data storage, data analytics, and machine learning functionalities. The component thought from Google for adding IoT functionalities to its platform is the so-called *Google Cloud IoT Core* [58], which augments the Google Cloud platform with heterogeneous devices connection and management. Google Cloud IoT Core, together with all the other components from the Google Cloud platform, allows an intelligent, versatile, and scalable management of IoT ecosystems. Among such components, fundamental for IoT are: (i) the *Cloud Dataflow*, which provides streaming and batch analytics functions and refers to the IoT Process Layer; (ii) *Cloud Pub/Sub*, which manages connections and communications so enhancing the communication layer; (iii) *Big Query*, which allows performing data warehouse and fast querying on the data acquired; and (iv) the *Cloud ML Engine* for training, deploying, and running machine learning models on the IoT data. The last two points excellently support the IoT service layer.

To date, Google Cloud IoT Core is used in a wide range of application fields. The main ones are predictive maintenance, real-time tracking, and logistics. Moreover, it is exploited for the realization of smart city applications that span from smart energy to smart parking and smart transportation.

#### Security in Google Cloud IoT Core

Google Cloud IoT Core supports five main security features [59]: (i) per-device public/private key authentication using JWTs (RFC 7519), aimed to limit the surface area of an attack, because a compromised key would affect only a single device and not the whole fleet and JWTs are valid for a limited duration, so any compromised keys will expire; (ii) RSA or elliptic curve algorithms to verify signatures, with enforcement for strong key sizes; (iii) key rotation per device by allowing concurrent keys to be registered, and support for expiration time per credential; (iv) TLS v.1.2 connection, using root certificate authorities, as required for MQTT; (v) IAM roles and permissions to control Cloud IoT Core API access.

### 3.11. Cumulocity IoT

Cumulocity IoT [60] is an interesting IoT platform built from scratch with the aim to be open, rapid to deploy, and distributed. Several heterogeneous devices can be connected to the platform in minutes and transparently used. It has been implemented to control IoT data and devices in (almost) real-time and without the need to implement code. Cumulocity IoT offers, so as to support the IoT service and the IoT process layers, a set of analytics and smart rules specifically implemented for business users as well as full developer tools for the more advanced users. Moreover, it has been developed to help in the connection of diverse applications and to synchronize data among them through several pre-built connectors. It works with lots of heterogeneous cloud apps, including SAP and Microsoft.

Cumulocity IoT is at the basis of several applications including, among others, supply chain automation, smart transportation, and smart agriculture.

#### Security in Cumulocity IoT

Cumulocity IoT addresses security on various levels by individual authentication and authorization methods [61]. Connections from and to Cumulocity IoT are established using HTTPS technology and all tenants have full rights to add or terminate users and user groups. At the physical layer, Cumulocity IoT Standard tenant accounts are hosted at AWS that has been certified according to ISO 27001 by featuring extensive physical security measures and is independently audited. However, since the operator of the Cumulocity IoT platform does not control the internal systems of their tenants, they must follow a powerful and carefully considered security concept for their own systems.

Cumulocity IoT ensures that your data stays confidential and cannot be tampered with through an end-to-end implementation of HTTPS from devices to applications. It uses up-to-date encryption technology and any communication with Cumulocity IoT is subject to individual authentication and authorization. It is recommended to use transport-level encryption (SSL/TLS) to protect all communications passing between the device and the platform; thus, the Strict-Transport-Security HTTP header should be used to ensure that devices refuse to access the platform over an insecure connection.

Cumulocity IoT uses a standard authentication and authorization process based on realms, users, user groups, and authorities. It also allows setting global permissions that are applicable to all managed objects, measurements, events, and so forth.

### 3.12. Oracle IoT Cloud Service

Oracle Cloud [62] is a very interesting platform of public cloud services enabling customers to build and run a wide range of different applications in a scalable, secure, highly available, and high-performance environment. Among the Oracle Cloud products, the most significant ones include the execution of advanced analytics, unified databases, and the code-less application development platform.

Oracle IoT Cloud Service [63] has the aim to simplify the IoT so allowing to rapidly assimilate IoT devices into any digital strategy and create novel IoT services, so supporting the IoT service layer. Oracle IoT Cloud Service wants to permit the integration of existing applications with IoT in an easy and efficient way. Moreover, Oracle IoT Cloud Service provides, for the IoT process layer, real-time analysis tools that allow correlations, aggregations, and filtering of any kind of IoT data. It also provides built-in integration with other Oracle services. Oracle IoT Cloud Service carefully adopts secure and reliable communications.

Regarding its most common application fields, Oracle IoT Cloud Service is used to monitor production facility performance and the health of production equipment in the smart industry. Moreover, it is the basis of several logistics and healthcare applications.

#### Security in Oracle IoT Cloud Service

Oracle IoT Cloud Service assigns a unique digital identity to each device to establish trust relationships among devices and applications. It also enforces authentication and authorization for end-to-end communication security and ensures proof of origin of data. During the authentication phase, the client asserts its identity using a JWT that can be signed using a secret (with HMAC algorithm) or a public/private key pair using RSA asymmetric cryptography algorithm.

## 4. Summary and Discussion

Some main considerations summarize the analysis of the surveyed IoT platforms:

1. All the listed IoT platforms provide advanced asset inter-connectivity solutions at the communication layer (mostly, through MQTT, HTTP, REST, OPC, and secured APIs) as well as proprietary/third-party analytic and visualization tools at the IoT process layer, as highlighted in Table 1. In particular, since inter-connectivity issues are a major complexity driver (protocol translation takes up a majority of today’s IoT development efforts), most IoT platforms are built around a standardized ecosystem;
2. There are several cloud-based IoT platforms adopting, mainly for the sake of usability, IaaS, PaaS, and SaaS solutions at the management layer to ensure horizontal/vertical scalability, automatic scaling, multizone backup, and auto load balancing;
3. There is an increasing general interest for open-source solutions/strategies, aimed at lowering cost and interoperability barriers, at all the architectural layers (e.g., Vorto for Bosch and PTC);
4. As Table 2 shows, the provided mechanisms at the security layer are typically limited to conventional cryptographic protocols (i.e., HTTPS, TLS) and vendor-specific policy, with only IIoT platforms deserving particular attention to cyber-security aspects;
5. End-users are most satisfied with the IoT platform’s capabilities, ease of use, and scalability of big cloud companies, especially for AWS and Azure, and most of them report positive IoT platform ROI [64].

Besides the above summary about the main features of the reviewed IoT platforms, some considerations can be drawn to highlight their limits. First of all, additional security methods need to be developed to protect the final solution by taking advantage of other selection criteria, like third-party and extended protocol support. Then, the majority of IoT platforms are cloud-based, few of them support hybrid cloud or edge computing architectures, although the benefits of decentralized paradigms (especially for the IoT service, security, and management layers) are undoubted, especially for implementing edge Intelligence solutions [65,66]. Moreover, apart from a few IoT platforms, there is poor attention to the modeling tools and, as result, rarely the digital twins or similar virtual alias are fully supported. Even more important, the lack of trustworthiness and the poor information about security mechanisms provided by some IoT platforms can be considered as a relevant issue for their adoption. Overall, in the review done, it is clear that all the platforms have many points in common regarding the functional features and, interestingly, non-functional characteristics (such as scalability, interoperability support, and end-to-end security) represent one of the main criteria for their discrimination.

Finally, please note that in our analysis we focused on security solutions provided at various layers of IoT platforms (especially at communication, service, and management ones like secure communication channels to avoid intrusions such as man-in-the-middle attacks; data encryption solutions to ensure data are safely encrypted while in transit and at rest; firewalls and intrusion prevention systems as foreseen by ISO to detect unwanted intrusions and prevent malicious activities; digital certificates for identification and authentication so as to verify the integrity of other cloud platforms or third party applications; policy enforcement, regular auditing, remote control, and security updates dispatching) but we did not deal with security features related to device’s hardware and software. To this extent, some manufacturers are introducing chip security in the form of trusted platform modules that act as a root of trust by protecting sensitive information and credentials (i.e., not releasing encryption keys outside the chip) [67]; secure booting to ensure only verified software will run on the device; physical security protection (e.g., full metal shield covering all internal circuitry) to guard against tampering if an intruder gains physical access to the device [68]. For example, in Microsoft Azure, device IDs can be associated with a device during manufacturing (that is, flashed in a hardware trust module) or can use an existing fixed identity as a proxy (for example CPU serial numbers). However, these topics about security features related to a device’s hardware and software are external to IoT platforms and, therefore, outside of the scope of this paper.

## 5. Conclusions

Currently, more than six hundred IoT platforms with different goals, targets, and functionalities have been successfully proposed to simplify the development of IoT ecosystems. Consequently, however, a relevant entry barrier for their selection and adoption has been raised due to the chaotic abundance of information sources (papers, websites, forums, project deliverables, etc.) and the redundancy of introduced solutions (e.g., tens of architectures, protocols, and standards).

Aiming to overcome such obstacles, the performed review has provided an hands-on state-of-the-art analysis of IoT platforms for the sake of both beginners and experts. In particular, we overviewed their key features through the lens of the high-level conceptual framework IoT-A RA, which consists of six layers, i.e., communication, virtual entity, IoT service, IoT process, management, and security. For each IoT platform, a focus has been given to the latter layer since ensuring the integrity, confidentiality, and protection against cybersecurity risks is no more considered an afterthought or an optional.

As a main consideration, we found that although baseline, fine-grained access control, and authentication mechanisms have been already implemented, further research and implementations at the security layer are still necessary, especially to support device/activity discovery and prevent information leakage and general vulnerabilities.

The future aim of the authors of this review is to try the reviewed platforms in the field so as to be more detailed in their comparison.

## Figures and Tables

**Table 1:** Analysis of main IoT platforms according to the IoT-A RA (✓basic, ✓✓advanced, ✓✓✓peculiar feature).

	Communication Layer	Virtual Entity Layer	IoT Service Layer	IoT Process Layer	Security Layer	Management Layer	Overall Score
**MindSphere**	✓✓	✓✓	✓✓	✓✓	✓	✓✓	✓✓
**IBM Watson**	✓✓	✓✓✓	✓✓	✓✓✓	✓	✓✓	✓✓
**Amazon AWS**	✓✓	✓	✓✓✓	✓✓	✓	✓✓✓	✓✓✓
**SAP IoT**	✓✓	✓✓	✓✓	✓✓✓	✓	✓✓✓	✓✓
**PTC ThingWorx**	✓✓	✓✓	✓✓	✓✓✓	✓✓	✓✓	✓✓✓
**Microsoft Azure**	✓✓	✓✓✓	✓✓	✓✓✓	✓✓	✓✓	✓✓✓
**Bosch IoT Suite**	✓✓✓	✓✓✓	✓✓	✓✓	✓	✓✓	✓✓
**GE Predix**	✓✓✓	✓✓	✓✓	✓✓	✓✓	✓✓	✓✓
**Hitachi Lumada**	✓✓	✓✓✓	✓✓	✓✓✓	✓✓	✓✓	✓✓
**Google IoT Core**	✓✓	✓	✓✓✓	✓✓	✓	✓✓	✓✓✓
**Cumulocity IoT**	✓✓	✓	✓✓✓	✓✓✓	✓	✓✓	✓✓
**Oracle IoT Cloud Service**	✓✓	✓	✓✓✓	✓✓	✓	✓✓	✓✓✓

**Table 2:** Security features.

Platform	Certification	Identity Management	Communication & Authentication	Unique Identification Number
**MindSphere**	ISO 27001	JSON Web Token (JWT)	HTTPS - TLS v. 1.2 - x509 certificate	(supported) MindConnect embedded UIN
**IBM Watson**	ISO 27001		HTTPS - TLS v. 1.2 - x509 certificate	(supported) IBM identification code
**AWS IoT**	ISO 27001	IAM (Identity and Access Management)	HTTPS - TLS v. 1.2 - x509 certificate	
**SAP IoT**	ISO 27001		HTTPS - TLS v. 1.2 - x509 certificate	
**PTC ThingWorx**		Active Directory	TLS	
**Azure IoT**	ISO 27001	Azure Active Directory (AAD)	HTTPS - TLS v. 1.2 - x509 certificate	(supported)
**Bosh IoT Suite**	ISO 27001	auth-id as part of the credentials	HTTPS; TLS v. 1.2 or DTLS; x509 certificate; PSK (pre-shared-key)	(supported)
**GE Predix**	ISO 27001/2 NIST 800-53 FIPS 140-2		TLS	
**Hitachi Vantara Lumada**	ISO 27001		HTTPS; TLS v. 1.2; x509 certificate	
**Google Cloud IoT Core**	ISO 27001/2 NIST 800-53	IAM (Identity and Access Management)	HTTPS - TLS v. 1.2 - x509 certificate	(supported)
**Cumulocity IoT**	ISO 27001		HTTPS TLS	
**Oracle IoT Cloud Service**		JSON Web Token (JWT)		(supported)

## Data Availability

Not applicable.

## References

[1] Fortino G., Savaglio C., Spezzano G., Zhou M. (2020). Internet of Things as System of Systems: A Review of Methodologies, Frameworks, Platforms, and Tools. IEEE Trans. Syst. Man Cybern. Syst..

[2] Guerrieri A., Loscri V., Rovella A., Fortino G. (2016). Management of Cyber Physical Objects in the Future Internet of Things.

[3] Nord J.H., Koohang A., Paliszkiewicz J. (2019). The Internet of Things: Review and theoretical framework. Expert Syst. Appl..

[4] Babun L., Denney K., Celik Z.B., McDaniel P., Uluagac A.S. (2021). A survey on IoT platforms: Communication, security, and privacy perspectives. Comput. Netw..

[5] Zdravković M., Trajanović M., Sarraipa J., Jardim-Gonçalves R., Lezoche M., Aubry A., Panetto H. Survey of Internet-of-Things platforms. Proceedings of the 6th International Conference on Information Society and Technology, ICIST 2016.

[6] Ray P.P. (2016). A survey of IoT cloud platforms. Future Comput. Inform. J..

[7] Dong Y., Yao Y.D. (2021). IoT platform for COVID-19 prevention and control: A survey. IEEE Access.

[8] Martín-Lopo M.M., Boal J., Sánchez-Miralles Á. (2020). A literature review of IoT energy platforms aimed at end users. Comput. Netw..

[9] Badii C., Belay E.G., Bellini P., Marazzini M., Mesiti M., Nesi P., Pantaleo G., Paolucci M., Valtolina S., Soderi M. Snap4city: A scalable iot/ioe platform for developing smart city applications. Proceedings of the 2018 IEEE SmartWorld, Ubiquitous Intelligence & Computing, Advanced & Trusted Computing, Scalable Computing & Communications, Cloud & Big Data Computing, Internet of People and Smart City Innovation (SmartWorld/SCALCOM/UIC/ATC/CBDCom/IOP/SCI).

[10] Mezquita Y., Casado R., Gonzalez-Briones A., Prieto J., Corchado J.M. (2019). AETiC. Blockchain technology in IoT systems: Review of the challenges. Annals of Emerging Technologies in Computing (AETiC).

[11] Ning H., Li Y., Shi F., Yang L.T. (2020). Heterogeneous edge computing open platforms and tools for internet of things. Future Gener. Comput. Syst..

[12] Frustaci M., Pace P., Aloi G., Fortino G. (2018). Evaluating Critical Security Issues of the IoT World: Present and Future Challenges. IEEE Internet Things J..

[13] Analytics I. (2021). IoT Platforms Market Report 2021–2026. https://iot-analytics.com/product/iot-platforms-market-report-2021-2026.

[14] Hejazi H., Rajab H., Cinkler T., Lengyel L. Survey of platforms for massive IoT. Proceedings of the 2018 IEEE International Conference on Future IoT Technologies (Future IoT).

[15] Erhan L., Ndubuaku M., Di Mauro M., Song W., Chen M., Fortino G., Bagdasar O., Liotta A. (2020). Smart anomaly detection in sensor systems: A multi-perspective review. Inf. Fusion.

[16] Sruthi M., Kavitha B. (2016). A survey on iot platform. Int. J. Sci. Res. Mod. Educ..

[17] Singh K.J., Kapoor D.S. (2017). Create Your Own Internet of Things: A survey of IoT platforms. IEEE Consum. Electron. Mag..

[18] Perry M.J. (2016). Evaluating and Choosing an IoT Platform.

[19] Ullah M., Smolander K. Highlighting the key factors of an IoT platform. Proceedings of the 2019 42nd International Convention on Information and Communication Technology, Electronics and Microelectronics (MIPRO).

[20] Bhatia A., Yusuf Z., Ritter D., Hunke N. (2017). Who Will Win the IoT Platform Wars?. BCG Perspectives.

[21] Pace P., Gravina R., Aloi G., Fortino G., Fides-Valero K., Ibanez-Sanchez G., Traver V., Palau C.E., Yacchirema D.C. IoT platforms interoperability for active and assisted living healthcare services support. Proceedings of the 2017 Global Internet of Things Summit (GIoTS).

[22] Fahmideh M., Zowghi D. (2020). An exploration of IoT platform development. Inf. Syst..

[23] Guth J., Breitenbücher U., Falkenthal M., Leymann F., Reinfurt L. Comparison of IoT platform architectures: A field study based on a reference architecture. Proceedings of the 2016 Cloudification of the Internet of Things (CIoT).

[24] Guth J., Breitenbücher U., Falkenthal M., Fremantle P., Kopp O., Leymann F., Reinfurt L. (2018). A Detailed Analysis of IoT Platform Architectures: Concepts, Similarities, and Differences. Internet of Everything: Algorithms, Methodologies, Technologies and Perspectives.

[25] Bassi A., Bauer M., Fiedler M., Kramp T., Van Kranenburg R., Lange S., Meissner S. (2013). Enabling Things to Talk.

[26] Fernandez E.B. Reference Architectures for the IoT: A Survey. Proceedings of the International Conference of Reliable Information and Communication Technology.

[27] Fortino G., Guerrieri A., Savaglio C., Spezzano G. A Review of Internet of Things Platforms through the IoT-A Reference Architecture. Proceedings of the 14th International Symposium on Intelligent Distributed Computing, IDC 2021.

[28] Frustaci M., Pace P., Aloi G. Securing the IoT world: Issues and perspectives. Proceedings of the 2017 IEEE Conference on Standards for Communications and Networking (CSCN).

[29] Analytics I. (2021). Global IoT Spending to Grow 24% in 2021, Led by Investments in IoT Software and IoT Security. https://iot-analytics.com/2021-global-iot-spending-grow-24-percent/.

[30] Analytics I. (2021). IoT Platform Companies Landscape 2021/2022: Market Consolidation Has Started. https://iot-analytics.com/iot-platform-companies-landscape/.

[31] (2021). MindSphere. https://siemens.mindsphere.io/en.

[32] IBM Watson. https://www.ibm.com/watson.

[33] (2021). Watson IoT Platform. https://www.ibm.com/cloud/watson-iot-platform.

[34] (2021). IBM Watson Security. https://www.ibm.com/docs/en/watson-iot-platform?topic=reference-security.

[35] (2021). Amazon Web Services. https://aws.amazon.com/.

[36] Wittig M., Wittig A. (2018). Amazon Web Services in Action.

[37] (2021). The Leading Cloud Providers Continue to Run Away with the Market. https://www.srgresearch.com/articles/leading-cloud-providers-continue-run-away-market/.

[38] (2021). AWS IoT. https://aws.amazon.com/iot/.

[39] (2021). Amazon Web Services Security Document. https://docs.aws.amazon.com/iot/latest/developerguide/iot-security.html.

[40] (2021). SAP. https://www.sap.com/.

[41] (2021). SAP Internet of Things. https://www.sap.com/products/iot-data-services.html.

[42] (2021). SAP Security Document. https://help.sap.com/viewer/2903b4da5b77448498f36d5769803776/2110b/en-US/a0ded3c2dab84658ae833842961bffae.html.

[43] (2021). ThingWorx. https://www.ptc.com/en/products/thingworx.

[44] (2021). About PTC. https://www.ptc.com/en/about.

[45] (2021). Microsoft Azure. https://azure.microsoft.com/.

[46] Copeland M., Soh J., Puca A., Manning M., Gollob D. (2015). Microsoft Azure.

[47] Azure IoT. https://azure.microsoft.com/en-us/overview/iot/.

[48] Klein S. (2017). IoT Solutions in Microsoft’s Azure IoT Suite.

[49] (2021). Bosch IoT Suite. https://bosch.io/iot-technology/.

[50] GE Predix. https://www.ge.com/digital/iiot-platform.

[51] Morris H.D., Ellis S., Feblowitz J., Knickle K., Torchia M. (2014). A software platform for operational technology innovation. Int. Data Corp..

[52] (2021). GE Predix Security Document. https://www.predix.io/resources/security.

[53] (2021). Hitachi Vantara Lumada. https://www.hitachivantara.com/en-us/products/iot-software-solutions/lumada-software-for-iiot.html.

[54] Kudo Y., Miyoshi T., Shimizu K., Takai M., Taguchi H. (2020). Lumada Solution Hub for Accelerated Development and Deployment of Digital Solutions. Hitachi Rev..

[55] (2021). Hitachi Vantara. https://www.hitachivantara.com/en-us/home.html.

[56] (2021). Lumada Security Document. https://help.hitachivantara.com/Documentation/Lumada/Lumada_Data_Catalog/Install/SSL_configuration.

[57] (2021). Google Cloud. https://cloud.google.com/.

[58] (2021). Google Cloud IoT Core. https://cloud.google.com/solutions/iot/.

[59] (2021). Google Cloud IoT Core Security. https://cloud.google.com/iot/docs/concepts/device-security.

[60] (2021). Cumulocity IoT. https://www.softwareag.cloud/site/product/cumulocity-iot.html#/.

[61] (2021). Cumulocity IoT Security Document. https://cumulocity.com/guides/concepts/security/.

[62] (2021). Oracle Cloud. https://www.oracle.com/cloud/.

[63] (2021). Oracle IoT Cloud Service. https://docs.oracle.com/en/cloud/paas/iot-cloud/.

[64] Houston C., Gooberman-Hill S., Mathie R., Kennedy A., Li Y., Baiz P. (2017). Case Study for the Return on Investment of Internet of Things Using Agent-Based Modelling and Data Science. Systems.

[65] Pace P., Fortino G., Zhang Y., Liotta A. (2019). Intelligence at the Edge of Complex Networks: The Case of Cognitive Transmission Power Control. IEEE Wirel. Commun..

[66] Savaglio C., Fortino G. (2021). A simulation-driven methodology for IoT data mining based on edge computing. ACM Trans. Internet Technol. (TOIT).

[67] Faisal M., Ali I., Khan M.S., Kim S.M., Kim J. (2020). Establishment of Trust in Internet of Things by Integrating Trusted Platform Module: To Counter Cybersecurity Challenges. Complexity.

[68] Cheruvu S., Kumar A., Smith N., Wheeler D.M. (2020). Demystifying Internet of Things Security: Successful Iot Device/Edge and Platform Security Deployment.

